# Correlation analysis between vaginal microecology and high-risk human papillomavirus (HR-HPV)-positive cervical squamous intraepithelial lesions (SIL)

**DOI:** 10.1097/MD.0000000000042914

**Published:** 2025-07-04

**Authors:** Baojin Zeng, Xiuling Ren, Yali Cheng, Chunhua Wang, Jiawei Li, Li Jiang, Shuai Zhang, Sisi Chen, Danjun Yu, Jingjing Lin

**Affiliations:** a Department of Gynecology, Taizhou Hospital of Zhejiang Province, Linhai City, Zhejiang Province, China; b Department of Gynecology, En Ze Medical Center (Group), En Ze Hospital, Taizhou City, Zhejiang Province, China; c Department of Inspection Center, The First Hospital of Qinhuangdao City, Qinhuangdao City, Shandong Province, China; d Department of Gynecology, The First Hospital of Qinhuangdao, Qinhuangdao City, Shandong Province, China; e Service Centre, Taizhou Hospital of Zhejiang Province, Linhai City, Zhejiang Province, China.

**Keywords:** high-grade squamous intraepithelial lesions, high-risk human papillomavirus, low-grade squamous intraepithelial lesions, vaginal microecology

## Abstract

This study is aimed to investigate the correlation between vaginal microecology and high-risk human papillomavirus (HR-HPV)-positive cervical squamous intraepithelial lesions (SIL) using the regression analysis. Patients (n = 372) with HR-HPV-positive from January 2020 to June 2022 were recruited after preliminary confirmation by colposcopy, HPV test, and typing, as well as loop electrosurgical excision procedure. Based on the pathological results, the recruited subjects were divided into 3 groups, that is, negative for intraepithelial lesion or malignancy, low-grade SIL, and high-grade SIL (HSIL). Finally, the clinical factors, virological data, and vaginal microecological changes of the 3 experimental groups were analyzed. Age was identified as a significant risk factor for HSIL, with an OR of 1.048 (95% CI: 1.006–1.094 and *P* = .026). Various HR-HPV types (HPV16, HPV18, and HPV52) were closely associated with HSIL, with multiple infections significantly increasing the risk (odds ratio, OR: 5.810, *P* = .04). The changes in the vaginal microecology were strongly associated with HSIL, including elevated pH (>4.5), reduced hydrogen peroxide levels, and increased bacterial vaginosis (BV) prevalence. BV demonstrated a sensitivity of 66.10% and a specificity of 70.31% for predicting HSIL. Furthermore, decreased *Lactobacillus* levels (OR: 3.20, *P* < .001) showed their protective role, while elevated sialidase activity (OR: 5.610, *P* = .002) emerged as a significant risk factor. Accordingly, the key independent predictors for low-grade SIL and HSIL included age, infection type, pH, microbiome density, BV, and sialidase activity. The mixed infection of HPV16, HPV18, HPV52, and HPV resulting in cervical SILs could be closely related to the vaginal microecology.

## 1. Introduction

The persistent human papillomavirus (HPV) infection has emerged as one of the most dreadful invasive cervical cancers.^[1]^ Due to the limited preventive vaccination, HPV infection often affects several women in developing countries every year, accounting for numerous deaths globally.^[1]^ Previous studies reported that vaginal microecology could be substantially associated with the clinical outcome of HPV infection, that is, HPV clearance, regression of squamous intraepithelial lesions (SIL), persistent HPV infection, or progression to precancerous intraepithelial neoplasia or even invasive cervical cancers.^[2–6]^ According to the degree of HPV association with precancerous lesions, HPV-related infections are broadly classified into high-risk HPV (HR-HPV) and low-risk HPV. The common HR-HPV types include HPV16, 18, 26, 31, 33, 35, 39, 45, 51, 52, 53, 56, 58, 59, 66, 68, and 73. To this end, the low-risk types include HPV6, 11, 40, 42, 43, 44, 81, and 83. The simultaneous infection with one or more different types of notified HPV strains is also evident, which can be further classified into single subtype infection and mixed infection with several subtypes (including superinfection, triple infection, and more subtypes). Among the mixed infection types, dual infection is most commonly seen clinically. However, it remains debatable whether the mixed infection with multiple HPV subtypes promotes the occurrence and development of cervical lesions.

Without any infection in the reproductive system, the vaginal microecology of healthy women is often dominated by different lactobacilli species.^[7,8]^ Previous reports indicated that women infected with HPV showed significantly reduced amounts of lactobacilli and increased bacterial diversities in the vaginal microecology than in the women without HPV.^[9–11]^ In addition, no significant difference between HPV-positive and HPV-negative women was observed regarding the bacterial diversity.^[12,13]^ Reportedly, significant changes were observed in the vaginal microecology of women with cervical SIL irrespective of HPV infection.^[9,12,14]^ Furthermore, the vaginal microecology of different lactobacilli species could produce altered clinical outcomes for HPV-positive women.^[15–17]^ For instance, it was demonstrated that women with vaginal microecology dominated by *L. crispatus* were positively correlated with the natural clearance of preexisting HPV infection. Nevertheless, the risk of persistent HPV infection was increased in the case of other vaginal microecologies.^[15,17]^ The impact of HPV infection on clinical outcomes could be attributed to the resultant immune responses,^[18,19]^ which might be influenced by ethnicity and lifestyle.^[20,21]^ Although several reports analyzed the differences in vaginal microecology between HPV-negative and HR-HPV-positive women, some disagreement existed regarding the correlation between the vaginal microecology and different degrees of SIL caused by HR-HPV infection. Moreover, traditional methods of analyzing these correlations are often limited by their inability to handle complex, multidimensional datasets effectively. Recent advancements in machine learning have demonstrated significant potential in analyzing clinical and microbiological data to discover hidden patterns and improve diagnostic accuracy. Several techniques, such as feature selection, dimensionality reduction, and predictive modeling, have been applied in diverse medical contexts, including cancer prediction, infection analysis, and biomechanical evaluations.^[22,23]^

Motivated by these aspects, this study is aimed to explore and understand the correlation and differences in vaginal microecology among patients with benign changes in their cervical tissues, the low-grade SIL (LSIL), and the high-grade SIL (HSIL) groups infected with HR-HPV. Further, we explored the relationship between vaginal microecology and SIL caused by HR-HPV infection, establishing new ideas and strategies for preventing and treating cervical cancer.

## 2. Materials and methods

### 2.1. Subjects

For this study, female patients (n = 372) with HR-HPV-positive infection status who visited our hospital between January 2020 and June 2022 were recruited. The positive infection status was confirmed by colposcopy, HPV testing, and typing, vaginal microecological balance status testing, cytology, as well as loop electrosurgical excision procedure. According to the cervical pathological findings, the recruited subjects were divided into 3 groups, including the group without intraepithelial lesions and malignant cells, referred to as negative for intraepithelial lesion or malignancy (NILM), LSIL, and HSIL. During the recruitment of subjects, the specific criteria were followed. On the one hand, the inclusion criteria for recruiting patients were set, including women older than 18 years of age, with normal sexual life, HPV testing positive, and high-risk type. On the other hand, the exclusion criteria were set as follows: patients who were pregnant and under lactating conditions; patients who were in postmenopausal condition; patients who had a history of cervical resection or hysterectomy or pelvic radiotherapy; patients who were taking immunosuppressive agents and oral antibiotics, and vaginal irrigation; patients who were having sex in the week before colposcopy. After recruitment, the data on relevant clinical factors were collected from all the patients, including age, fertility history, amount of smoking, contraception, and human immunodeficiency (HIV) infection. This study was approved by the ethics committee of our hospital. Notably, a written informed consent was signed by the patients before collecting all the samples and data. It should be noted that the collected information was secured such that none of the authors or participants had access to identify individual participants during or after data collection.

### 2.2. Methods

#### 2.2.1. Sample collection

Briefly, the subject was initially advised to take the cystolithotomy position on the examination bed. After cleaning the excess lubrication with sterile paraffin cotton balls, a sterile speculum was slowly placed into the vagina. Then, the secretions were collected from the fully exposed cervix in the upper 1/3 area of the vagina and cleaned with special sterile cotton swabs for vaginal microecological detection. Further, the speculum was placed in a special preservation tube and delivered within 30 minutes. The cervical exfoliated cells were collected by inserting a cervical brush deep (1–2 cm) into the cervical canal. After rotating the brush clockwise for 3 turns, the sampler brush tip was placed into a sampling bottle containing a liquid-based cell preservation solution. The workflow framework of this study is illustrated in Fig. S1, Supplemental Digital Content, https://links.lww.com/MD/P274. Based on pathological diagnosis, the datasets were comprised of 372 samples from HR-HPV-positive women categorized into NILM (192 samples), LSIL (118 samples), and HSIL (62 samples) groups. The key clinical and microbiological features were included for analysis, such as age, HR-HPV infection types, and vaginal microecological indicators. Each dataset contained a training set (75%, 279 samples) and a testing set (25%, 93 samples) to evaluate the model performance.

#### 2.2.2. HPV detection

The FDA-approved Cobas 4800 platform was applied to detect HPV (Roche Molecular Diagnostics, Pleasanton) in the collected cell samples. Each detection run included automated sample preparation, PCR amplification, and real-time fluorescence detection. Accordingly, eleven types of HR-HPV strains, such as HPV16, 18, 33, 35, 39, 45, 51, 52, 56, 58, and 66, were detected.

#### 2.2.3. Vaginal microecological balance examination

Several vaginal microecological indicators were investigated by microscopic examination of vaginal secretions and vaginal microecological detection instruments, such as pH value, the density, and diversity of flora, Candida, bacterial vaginosis (BV), Trichomonas, Lactobacillus, catalase, cleanliness, leukocyte esterase, and sialidase. Indeed, the typical pH value of the vaginal microecology was in the range of 3.8 to 4.5, the density of flora as class II to III, the diversity of flora as class II to III, the dominant bacteria as *Lactobacillus* species and normal hydrogen peroxide (H_2_O_2_) concentration. Moreover, no pathogenic microorganism was observed, and leukocyte esterase, sialidase, and catalase were all negative or normal.

#### 2.2.4. Cytological and pathological diagnosis

To determine the pathological diagnosis, a direct biopsy was performed for all visible lesions, and a random biopsy was performed at the squamoid junction in the normal quadrant. Briefly, the tissue samples were fixed in formalin and sectioned into thin slices at a thickness of 4 to 5 µm. These thin-sectioned tissues on slides were deparaffinized in xylene for approximately 10 minutes and rehydrated with alcohol and water (100%, 95%, 70%, and distilled water), with each step lasting approximately 2 minutes. Then, the slides were stained with hematoxylin for approximately 5 minutes, rinsed in running tap water, and differentiated in a 1% acid–alcohol mixture for 30 seconds. After rinsing, the tissue sections were counterstained with eosin for approximately 2 minutes. Afterward, the tissues were dehydrated through an ascending alcohol series and cleared in xylene for approximately 5 minutes. The prepared slides were examined under a light microscope at various magnifications (10× and 40×) to evaluate tissue architecture and cellular morphology. The highest grade from multiple biopsies in each quadrant was recorded as the final diagnosis. Notably, the pathological diagnosis was negative for cervical intraepithelial neoplasia (CIN) grades I, II, and III, or cancer. According to The Bethesda System grading scale, CIN I and CIN II/III were classified as LSIL and HSIL, respectively.

### 2.3. Statistical analysis

The detailed experimental data were expressed in terms of mean ± standard deviation and were analyzed by SPSS 23.0 statistical software using a *t* test. In addition, the counting data were expressed as a rate in terms of percentage (%). By applying the chi-square test, the logistic regression analysis was used to calculate or/and 95% CI to describe the risk of occurrence of related risk factors, considering a defined value of *P* < .05 as statistically significant.

## 3. Results

### 3.1. Comparison of relevant clinical factors

Initially, the data of patient-related clinical factors among the 3 groups (NILM, LSIL, and HSIL) of recruited subjects were compared, including age, smoking, parity, smoking, contraceptive usage, and HIV infection. As presented in Table 1, no statistically significant difference was observed in the fertility status of the patients irrespective of smoking, contraceptive measures taken, and co-infection with HIV. Among all the factors, the age of the patients showed a significant difference (*P* < .05) between those with and without pathological changes, indicating that age was a substantial risk factor for HSIL. The mean age of patients was 36.8 ± 4.2 years for the NILM group of subjects, 37.6 ± 7.2 years for the LSIL group of subjects, and 38.4 ± 8.1 years for the HSIL group of subjects (*P* < .05). Among these notified vaginal microecological indicators, the pH levels were significantly higher (*P* < .01) in the HSIL group of subjects (5.2 ± 0.6) compared to the NILM group of subjects (4.5 ± 0.4) and the LSIL group of subjects (4.8 ± 0.5).

**Table 1:** A summary shows the sociodemographic and virological data according to the severity of SIL during enrollment.

Parameter	NILM (n = 192)	LSIL (n = 118)	HSIL (n = 62)
Age (Year)	36.81 ± 4.21	37.61 ± 7.15	38.42 ± 8.08*
Parity (n, %)	78 (40.63)	46 (38.98)	25 (40.32)
Smokers (n, %)			
<20 cig/d	21 (10.93)	12 (10.17)	6 (9.68)
≥20 cig/d	24 (12.51)	13 (11.02)	7 (11.29)
Nonsmokers	147 (76.56)	93 (78.81)	49 (79.03)
Contraceptive usage (n, %)			
No	101 (52.6)	57 (48.31)	32 (51.61)
Barrier	16 (8.33)	9 (7.63)	5 (8.06)
Hormonal	57 (29.69)	33 (27.96)	18 (29.03)
IUD	15 (9.38)	19 (16.1)	7 (11.3)
HIV-positive (n, %)	8 (4.17)	4 (3.39)	3 (4.83)

HIV = human immunodeficiency virus, HSIL = high-grade squamous intraepithelial lesion, LSIL = low-grade squamous intraepithelial lesion, NILM = negative for intraepithelial lesion or malignancy.

* Indicates *P* < .05.

### 3.2. Distribution of biopsy findings and HR-HPV types

Further, the types of HPV infections were identified among patients in the 3 groups of subjects. As depicted in Table 2, various HPV strains were detected, including 209 strains in 192 patients of the NILM group of subjects, 146 strains in 118 patients of the LSIL group of subjects, and 114 strains in 62 patients of the HSIL group of subjects. Several HR-HPV types (HPV16, 18, 33, 35, 39, 45, 51, 52, 56, 58, and 66) were determined. Among these types, the proportion of patients in the HSIL group infected with HPV16 (33.33%), HPV18 (11.40%), and HPV52 (21.05%) was significantly higher compared to the NILM and LSIL groups (*P* < .05). Notably, HPV52 emerged as a common subtype, ranking second in prevalence after HPV16 in the HSIL group, emphasizing its potential role in the progression of high-grade lesions.

**Table 2:** A summary indicates the dependency between biopsy results and HPV types.

Type of infection	NILM (n = 209)	LSIL (n = 146)	HSIL (n = 114)
HPV16	51 (24.40%)	43 (29.45%)	38 (33.33%)*
HPV18	17 (8.13%)	12 (8.22%)	13 (11.40%)*
HPV33	12 (5.74%)	8 (5.48%)	5 (4.39%)
HPV35	14 (6.70%)	9 (6.16%)	4 (3.51%)
HPV39	19 (9.09%)	8 (5.48%)	5 (4.39%)
HPV45	15 (7.18%)	6 (4.11%)	6 (5.26%)
HPV51	28 (13.40%)	14 (9.59%)	8 (7.02%)
HPV52	22 (10.53%)	27 (18.49%)	24 (21.05%)*
HPV56	9 (4.31%)	5 (3.42%)	3 (2.63%)
HPV58	9 (4.31%)	7 (4.79%)	4 (3.51%)
HPV66	13 (6.22%)	7 (4.79%)	4 (3.51%)

HSIL = high-grade squamous intraepithelial lesion, LSIL = low-grade squamous intraepithelial lesion, NILM = negative for intraepithelial lesion or malignancy.

* indicates *P* < .05.

### 3.3. Correlation analysis between multiple HR-HPV infections and cervical SIL types

A correlation was established between multiple HR-HPV infections and cervical SIL types (Table 3). It was observed from the results that a single HPV infection was predominant in the NILM and LSIL groups of subjects, accounting for 73.68% and 61.64%, respectively. The proportion of patients infected with a single type of strain in these NILM and LSIL groups was significantly higher than the proportion of patients in the HSIL group (*P* < .05). Contrarily, amphotropic HPV or dual infection was predominant in the HSIL group, with a proportion of 58.77%. Similarly, the proportion of patients with two- and three-kinds of HPV infectious strains was significantly higher in the HSIL group than in the NILM and LSIL groups (*P* < .05). The diagnostic performance analysis revealed that 2 kinds of infectious strains showed a sensitivity rate of 58.77% and a specificity of 69.86%. In contrast, three kinds of infectious strains exhibited a sensitivity rate of 13.16% and a specificity of 87.51%.

**Table 3:** A summary presents the correlation analysis between multiple infections with HR-HPV and types of cervical SIL.

Type of infection	NILM (n = 209)	LSIL (n = 146)	HSIL (n = 114)
Single-infection	154 (73.68)	90 (61.64)	32 (28.07)*
Double-infection	65 (31.10)	45 (30.82)	67 (58.77)*
Triple- or more infection	10 (4.78)	11 (7.53)	15 (13.16)*

HSIL = high-grade squamous intraepithelial lesion, LSIL = low-grade squamous intraepithelial lesion, NILM = negative for intraepithelial lesion or malignancy.

* Indicates *P* < .05.

### 3.4. Variations in the vaginal microecologically dominant flora

**Figure 1.**
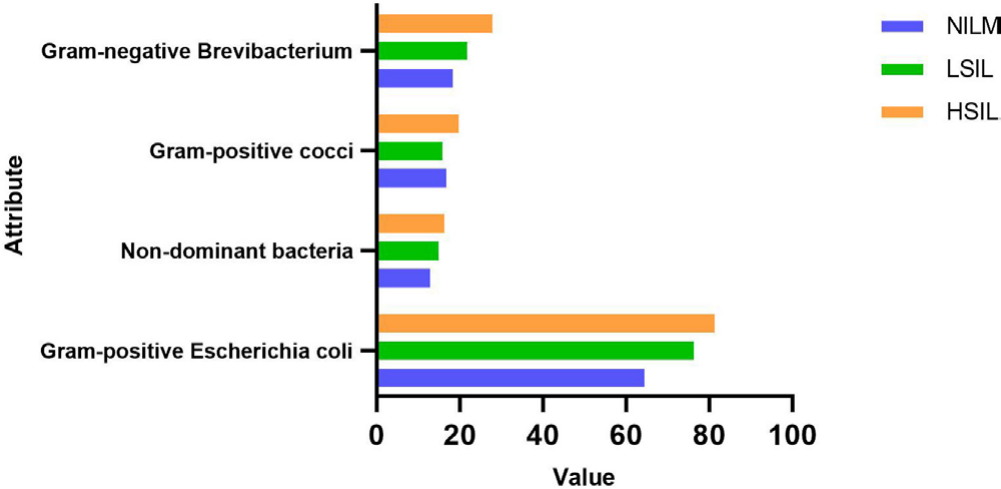
The image presents the status of dominant vaginal bacteria in the 3 groups of subjects.

Figure 1 shows the dominant flora in the vaginal microecology of the 3 treatment groups of patients (NILM, LSIL, and HSIL). Specifically, the vaginal microecology was dominated by gram-positive enterobacteria, gram-negative *Brevibacillus*, gram-positive cocci, and many other kinds of bacteria. Among the 3 groups, the vaginal flora diversity was more abundant in patients of the HSIL group compared to the other 2 groups (NILM and LSIL) of subjects. Nonetheless, the differences in the existence of dominant flora between them were not statistically significant.

### 3.5. Correlation analysis between vaginal microecological changes and types of cervical SILs

Using the regression analysis, a significant correlation between HSIL occurrence and various vaginal microecological factors (Table 4) was identified. A decrease in vaginal pH (OR: 2.85, 95% CI: 1.45–5.60, *P* = .01) and H_2_O_2_ levels (OR: 3.12, 95% CI: 1.78–5.92, *P* < .01) was strongly associated with HSIL. In addition, HSIL risk was significantly increased due to density and diversity of reduced flora (OR: 2.28, 95% CI: 1.34–4.25, *P* = .03), BV (OR: 3.70, 95% CI: 2.12–6.45, *P* < .001), and elevated sialidase activity (OR: 4.05, 95% CI: 2.25–7.42, *P* < .001). In addition, vaginal conditions showed strong associations, including aerobic vaginitis (OR: 2.95, 95% CI: 1.62–5.16, *P* = .02), trichomonas vaginitis (OR: 3.45, 95% CI: 1.85–6.24, *P* = .01), and cytolytic vaginosis (OR: 2.15, 95% CI: 1.11–4.12, *P* = .04). Moreover, the significantly reduced lactic acid bacteria levels (OR: 3.20, 95% CI: 1.88–5.65, *P* < .001) could determine the protective role of a *Lactobacillus*-dominant vaginal environment in preventing HSIL progression. As shown in Table 5, the pH value of >4.5 exhibited high sensitivity (81.36%) but moderate specificity (63.54%) for predicting HSIL. Similarly, BV showed a sensitivity of 66.10% and a specificity of 70.31%, highlighting its potential clinical relevance.

**Table 4:** A summary presents the association between the vaginal microenvironment changes and SIL in different HPV-infected groups.

Factors	HR-HPV group (n = 372)		
	Group (n)	Abnormal persons (n, %)	OR (95% CI)
pH	NILM (192)	122 (63.54)	1.00
	LSIL (121)	90 (74.38)	2.13 (0.81–3.63)
	HSIL (59)	48 (81.36)	3.31 (1.71–6.75)
	*χ*^2^ = 11.413, *P* < .001		
H_2_O_2_	NILM (192)	131 (68.23)	1.00
	LSIL (121)	98 (80.99)	2.09 (0.93–3.82)
	HSIL (59)	52 (88.14)	3.28 (1.03–7.11)
	*χ*^2^ = 15.673, *P* < .001		
Microbiome density	NILM (192)	98 (51.04)	1.00
	LSIL (121)	78 (64.46)	1.98 (0.79–3.58)
	HSIL (59)	44 (74.58)	3.42 (1.23–7.16)
	*χ*^2^ = 14.287, *P* < .001		
Microbiome diversity	NILM (192)	94 (48.96)	1.00
	LSIL (121)	79 (65.29)	2.04 (1.02–3.85)
	HSIL (59)	45 (76.27)	4.35 (1.37–7.86)
	*χ*^2^ = 17.119, *P* *<* .001		
BV	NILM (192)	57 (29.69)	1.00
	LSIL (121)	68 (56.20)	2.07 (0.95–3.18)
	HSIL (59)	39 (66.10)	3.57 (1.16–6.94)
	*χ*^2^ = 15.738, *P* *<* .001		
AV	NILM (192)	74 (38.54)	1.00
	LSIL (121)	63 (52.07)	2.31 (0.94–3.62)
	HSIL (59)	38 (64.41)	3.65 (1.18–6.49)
	*χ*^2^ = 16.114, *P* *<* .001		
Vulvovaginal candidiasis (VVC)	NILM (192)	55 (28.65)	1.00
	LSIL (121)	37 (30.58)	1.24 (0.64–1.83)
	HSIL (59)	20 (33.90)	1.75 (0.78–2.41)
	*χ*^2^ = 5.903, *P* = .083		
TV	NILM (192)	50 (26.04)	1.00
	LSIL (121)	46 (38.01)	1.45 (0.75–1.94)
	HSIL (59)	27 (45.76)	2.89 (0.81–3.47)
	*χ*^2^ = 9.328, *P* = .015		
CV	NILM (192)	107 (55.73)	1.00
	LSIL (121)	86 (71.07)	2.13 (0.81–1.63)
	HSIL (59)	50 (84.75)	3.09 (1.02–5.89)
	*χ*^2^ = 14.266, *P* < .001		
Leukocyte esterase	NILM (192)	118 (61.46)	1.00
	LSIL (121)	82 (67.77)	1.25 (0.64–1.79)
	HSIL (59)	41 (69.49)	1.31 (0.83–2.25)
	*χ*^2^ = 6.229, *P* = .062		
Sialidase	NILM (192)	8 (4.17)	1.00
	LSIL (121)	13 (10.74)	2.04 (1.12–3.48)
	HSIL (59)	9 (15.25)	3.11 (0.95–5.83)
	*χ*^2^ = 13.228, *P* < .001		
Catalase	NILM (192)	101 (52.60)	1.00
	LSIL (121)	63 (52.07)	1.19 (0.58–1.71)
	HSIL (59)	33 (55.93)	1.23 (0.72–1.97)
	*χ*^2^ = 4.276, *P* = .163		
*Lactobacillus*	NILM (192)	54 (28.13)	1.00
	LSIL (121)	49 (40.50)	1.74 (0.68–2.34)
	HSIL (59)	23 (47.46)	2.85 (0.84–4.01)
	*χ*^2^ = 9.453, *P* = .013		

pH > 4.5 considered abnormal, H_2_O_2_ = detected or undetected, microbiome density = class II to III categorized as abnormal, microbiome diversity = class II to III categorized as abnormal, BV = positive or negative, HR-HPV infection = single, double, or triple/more infection types.

95% CI: 95% confidence interval, AV = aerobic vaginitis, BV = bacterial vaginosis, CV = cytolytic vaginosis, H_2_O_2_ = hydrogen peroxide, HPV = human papillomavirus, HR-HPV = high-risk human papillomavirus, HSIL = high-grade squamous intraepithelial lesion, LSIL = low-grade squamous intraepithelial lesion, NILM = negative for intraepithelial lesion or malignancy, OR = odds ratio, pH = power of hydrogen, TV = trichomonas vaginitis, VVC = vulvovaginal candidiasis, χ² = Chi-square test.

**Table 5:** The data present the diagnostic performance for HSIL.

Variable	Sensitivity (%)	Specificity (%)
pH	81.36	32.27
H_2_O_2_	88.14	26.84
Microbiome density	74.58	43.77
Microbiome diversity	76.27	44.73
BV	66.1	60.06
AV	64.41	56.23

AV = aerobic vaginitis, BV = bacterial vaginosis.

### 3.6. Influence of various characteristics on the occurrence of LSIL or HSIL

Table 6 presents the univariate and multivariate analyses assessing the impact of various characteristics on the likelihood of LSIL or HSIL occurrence. Various factors demonstrated significant associations with LSIL or HSIL presence, including age, infection type, pH level, H_2_O_2_ concentration, microbiome density, BV, and other specific factors. In both analyses, the patient’s age consistently showed a positive correlation between LSIL/HSIL occurrence and the multivariate odds ratio (OR). These findings indicated that each additional year increased the odds by approximately 1.048 (95% CI: 1.006–1.094, *P* = .026). In addition, the infection type was a notable predictor. Specifically, individuals with triple or more infections exhibited a significantly higher risk, with an OR of 5.810 (95% CI: 1.284–42.67, *P* = .04) in the multivariate model than the single type. Similarly, the microbiome density and pH levels were strong predictors. Accordingly, these factors showed significantly elevated ORs in the multivariate analysis. The microbiome density and pH value showed an OR value of 2.983 (95% CI: 1.745–5.212, *P* < .001) and 2.777 (95% CI: 1.579–4.997, *P* < .001). In addition, various factors, including BV and sialidase activity, emerged as substantial contributors. BV presented an OR value of 3.691 (95% CI: 2.195–6.323, *P* < .001), while sialidase activity showed an OR value of 5.610 (95% CI: 1.998–17.83, *P* = .002). Contrarily, other factors, such as catalase activity and contraceptive usage, were not significantly associated with LSIL or HSIL in these univariate and multivariate analyses, as reflected by their nonsignificant *P*-values.

**Table 6:** The data present the impact of various characteristics on the occurrence of LSIL or HSIL in univariate and multivariate analyses, along with their statistical significance.

Characteristics	Uni-B	Uni-OR	Uni-CI	Uni-P	Multi-B	Multi-OR	Multi-CI	Multi-P
Age	0.041	1.041	1.041 (1.008–1.077)	.016	0.048	1.049	1.048 (1.006–1.094)	.026
Parity	‐0.177	0.837	0.837 (0.553–1.265)	.4				
Smokers								
≤20 cig/day								
≥20 cig/day	0.132	1.141	1.141 (0.595–2.201)	.691				
Nonsmokers	‐0.444	0.642	0.642 (0.283–1.441)	.283				
Contraceptive usage								
Barrier								
Hormonal	‐0.549	0.577	0.577 (0.231–1.414)	.231				
IUD	0.481	1.618	1.618 (0.602–4.33)	.336				
No	‐0.432	0.649	0.649 (0.276–1.492)	.31				
HIV	0.227	1.254	1.254 (0.409–3.966)	.689				
Infection type								
Double-infection								
Single-infection	‐0.768	0.464	0.464 (0.294–0.728)	.001	-0.801	0.449	0.448 (0.255–0.777)	.005
Triple- or more infection	1.71	5.529	5.529 (1.48–36.02)	.027	1.76	5.811	5.810 (1.284–42.67)	.04
pH	0.906	2.474	2.474 (1.575–3.934)	0	1.021	2.777	2.777 (1.579–4.997)	<.001
H_2_O_2_	0.743	2.102	2.102 (1.297–3.458)	.003	1.045	2.844	2.844 (1.552–5.357)	.001
Microbiome density	0.738	2.091	2.091 (1.371–3.211)	.001	1.093	2.984	2.983 (1.745–5.212)	<.001
Microbiome diversity	0.489	1.63	1.63 (1.082–2.465)	.02	0.319	1.376	1.375 (0.824–2.302)	.222
BV	1.226	3.409	3.409 (2.226–5.27)	0	1.306	3.691	3.691 (2.195–6.323)	<.001
AV	0.343	1.409	1.409 (0.937–2.122)	.1				
VVC	0.073	1.076	1.076 (0.699–1.656)	.739				
TV	0.757	2.131	2.131 (1.392–3.284)	.001	0.835	2.304	2.303 (1.360–3.950)	.002
CV	1.002	2.722	2.722 (1.75–4.286)	0	0.927	2.527	2.526 (1.484–4.365)	.001
Leukocyte esterase	0.686	1.985	1.985 (1.297–3.059)	.002	0.395	1.484	1.484 (0.869–2.541)	.148
Sialidase	1.25	3.491	3.491 (1.513–9.058)	.005	1.725	5.611	5.610 (1.998–17.83)	.002
Catalase	0.105	1.11	1.11 (0.737–1.675)	.617				
Lactobacillus	0.664	1.943	1.943 (1.269–2.991)	.002	0.7	2.014	2.013 (1.183–3.462)	.01

AV = aerobic vaginitis, BV = bacterial vaginosis, CV = cytolytic vaginosis, HIV = human immunodeficiency virus, VVC = vulvovaginal candidiasis, TV = trichomonas vaginitis.

## 4. Discussion

In recent times, it has been increasingly recognized that SIL and cervical cancer could be closely related to persistent HPV infection with its high-risk types.^[24]^ Consequently, HPV infection was mainly influenced by sexual activity, potentiating the infection in women with an altered vaginal microecology, suggesting vaginal microecological heterogeneity in HPV-positive women.^[13,25]^ In this context, vaginal microecology could be affected by several factors, such as hygiene habits, ethnicity, and age, explaining the inconsistent results of the vaginal microecological analysis in various cross-sectional studies.^[11,26]^ In this context, our study aimed to demonstrate a significant association between changes in vaginal microecology and the progression of HR-HPV-positive cervical SIL. In this context, we found that patients with HSIL exhibited altered vaginal microecology, including reduced *Lactobacillus* levels, elevated pH, and increased BV prevalence. In addition, multiple HR-HPV infections (e.g., HPV16, 18, and 52) were associated with a significant risk of HSIL, and these findings were in alignment with the reported studies.

Notably, HPV infection can orchestrate the spontaneous immune responses that, in turn, affect the vaginal microecology. Nevertheless, interactions between the vaginal microecology and the host are in place before HPV infection. Therefore, modulation of host responses by the vaginal microecological system significantly impacts the persistence of random HPV infection.^[27]^ In this vein, several reports demonstrated that there existed significant differences in the vaginal microecology between women who cleared their HPV infection and those with persistent infection.^[4,6,17]^ In addition, studies indicated that vaginal microecology showed an essential impact on the persistence of HPV infection.^[28]^ Accordingly, the clinical outcome of HPV infection might be determined by the vaginal microecology, which could function as a significant immunomodulator through the host–microbial interactions.

In our study, we highlight a significant correlation between HR-HPV subtypes, particularly HPV16, 18, and 52, and the progression of HSIL. Among the patients with HSIL, the prevalence of these 3 subtypes was notably higher than in the NILM and LSIL groups of subjects (*P* < .05). These findings were consistent with previously reported studies, identifying HPV16 and 18 as the most oncogenic subtypes that could account for approximately 70% of cervical cancers globally.^[29,30]^ Among Chinese women, HPV52 and HPV16 were identified to show the most significant impact on cervical health.^[31]^

Similarly, the present study indicated a correlation between multiple infections with HR-HPV and the type of cervical SIL.^[32]^ In addition, patients with 2 kinds of HPV infectious strains and 3 kinds of HPV infectious strains showed a higher risk of developing HSIL than a single type of HPV infectious strain. Our analysis revealed that 2 kinds of HPV infectious strains exhibited moderate sensitivity (58.77%) and specificity (69.86%), making them useful for initial screening. Despite their lowest sensitivity (13.16%), infection with 3 kinds of HPV infectious strains demonstrated the highest specificity (87.51%), indicating their suitability for confirmatory diagnostics. Nevertheless, it is still a rather controversial claim that mixed infection with multiple HPV subtypes promotes the occurrence and development of cervical lesions. In an instance, it was demonstrated that mixed infection with different HR-HPV strains resulted in HSIL. Moreover, the risk of HSIL from mixed infection with HR-HPV and HR-HPV was 3-fold higher than from single HR-HPV infection.^[33]^ Moreover, HPV persistent infection was prone to co-infection with other subtypes during various infections.^[29]^ In addition, the mixed HR-HPV infection conferred a higher risk of high-grade lesions than infection with monotypic HR-HPV.^[34]^ However, mixed HR-HPV infection could not increase the risk of cervical cancer and its precancerous lesions.^[35]^

Previous reports indicated that several anaerobes associated with BV, such as *Gardnerella* and *Prevotella* species, were present in women with SIL,^[9]^ affecting the viral infection persistence, as well as cervical disease initiation and progression through several cellular pathways.^[36,37]^ In a case, the genus *Leptotrichia* was predominantly accumulated in the vaginal microecology of BV patients, promoting carcinogenesis by activating pro-inflammatory pathways and inhibiting immune cytotoxicity.^[38]^ Accordingly, several reports indicated a positive correlation between BV and vaginal obligate anaerobe accumulation in women with HPV infection and progression of persistent HPV infection and cervical SIL. Similar to the above findings, the present study demonstrated that the diversity of the flora in patients in the HSIL group of subjects was significantly higher than that in the NILM group of subjects.

In our study, the vaginal microecology of patients in the HSIL group of subjects showed lower *Lactobacillus* content than in the other 2 groups of subjects (NILM and LSIL). Notably, the *Lactobacillus vaginalis* could inhibit the colonization of common pathogens associated with BV, such as *Chlamydia trachomatis*, *Gonococcus*, and *Gardnerella*. Moreover, lactobacilli could prevent biofilm formation by pathogenic anaerobes through excreted biosurfactants, inhibiting the overgrowth of these pathogens. Among them, *Prevotella* and *Gardnerella* showed a limited capacity to produce catalase-degrading enzymes, which could be inhibited by lactobacilli-associated production of H_2_O_2_.^[38]^ In addition to its direct inhibitory effects on pathogenic bacteria, lactic acid bacteria could offer indirect effects on preventing colonization by pathogenic bacteria. For example, epithelial adhesins could promote the adhesion of *L. crispatus* to the genital mucosa, thereby inhibiting the *Trichoderma*-mediated adhesion of *Gardnerella*.^[39,40]^ These findings could explain that altered vaginal microecology might significantly contribute to HSIL. However, the implementation of deep learning models for analyzing such complex relationships and predicting HSIL outcomes requires substantial computational resources. The cost associated with deploying these models, including high-performance hardware (e.g., GPUs or TPUs) and software platforms, can pose to resource-limited settings. To address this shortcoming, cloud-based platforms can be used, offering a scalable alternative and enabling institutions to leverage advanced computational tools without significant upfront investments. In addition, optimizing model architectures through techniques, including pruning and quantization, can reduce hardware requirements and operational costs, as well as improve accessibility and feasibility.

Although our findings provide valuable insights, several limitations need to be addressed with further investigation. The cross-sectional nature of the study may prevent establishing causality. In addition, the relatively small HSIL group sample size may limit statistical power for subgroup analyses. Finally, various vaginal microecological parameters, and other confounding factors, such as diet, hygiene practices, or hormonal influences, remain to be assessed.

## 5. Conclusion

In summary, our study has demonstrated the significant contributing factors to the development of HSIL, including age, infection with HPV16, 18, and 52 subtypes, and mixed infection with HR-HPV. In addition, the alteration of vaginal microecology showed a close relationship with the development of cervical lesions. Our findings suggested that the vaginal microbiome, along with *Lactobacillus* levels, pH, and bacterial vaginosis, played a significant role in the progression of HR-HPV-associated lesions. However, further research is needed to understand the mechanisms linking these microbiological changes to the pathogenesis of cervical lesions and to explore potential therapeutic strategies that target vaginal microbiome modulation. Future studies should focus on longitudinal investigations to establish causal relationships, as well as the development of microbiome-based biomarkers for early prediction and intervention in HPV-associated cervical diseases.

## Author contributions

**Conceptualization:** Jingjing Lin.

**Data curation:** Baojin Zeng, Jiawei Li.

**Formal analysis:** Jiawei Li.

**Investigation:** Baojin Zeng, Li Jiang, Danjun Yu.

**Methodology:** Baojin Zeng, Li Jiang.

**Project administration:** Xiuling Ren.

**Resources:** Shuai Zhang.

**Software:** Shuai Zhang.

**Supervision:** Sisi Chen.

**Validation:** Yali Cheng, Sisi Chen.

**Visualization:** Xiuling Ren, Danjun Yu.

**Writing – original draft:** Chunhua Wang.

**Writing – review & editing:** Baojin Zeng, Xiuling Ren, Yali Cheng, Danjun Yu, Jingjing Lin.

## Supplementary Material


