# Supplementary figures and images for: Correlation analysis between vaginal microecology and high-risk human papillomavirus (HR-HPV)-positive cervical squamous intraepithelial lesions (SIL)
Source: Medicine (Baltimore). 2025 Jul 4;104(27):e42914. doi: 10.1097/MD.0000000000042914 (PMC12237335; doi:10.1097/MD.0000000000042914)

**Figure S1***. The image presents the study workflow for HR-HPV-positive cervical SIL analysis.*


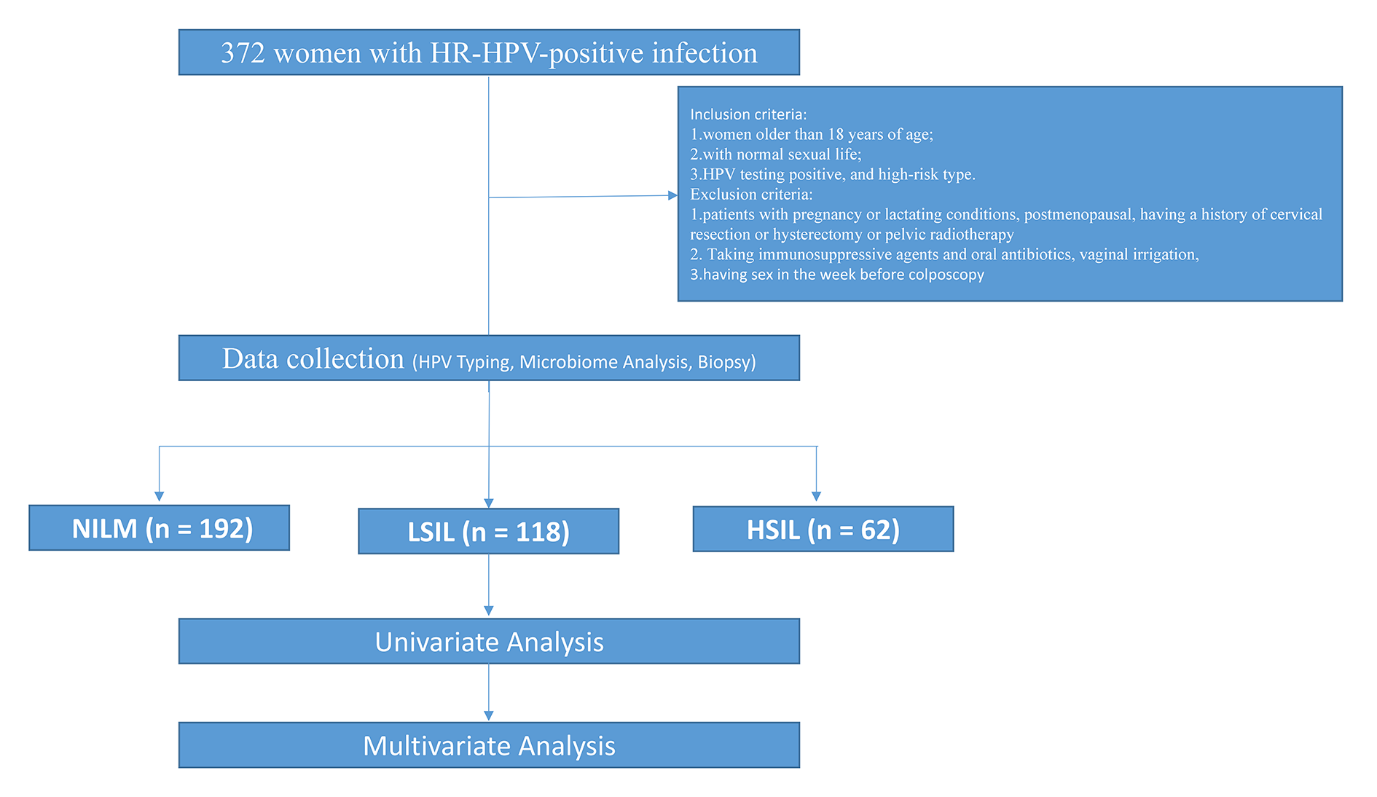

Supplement: Supplementary file 1 [file medi-104-e42914-s001.docx]
